# A case of hypothalamic hypopituitarism accompanied by recurrent severe hypoglycemia

**DOI:** 10.1186/s40064-015-0934-6

**Published:** 2015-04-10

**Authors:** Maiko Takai, Hideaki Kaneto, Shinji Kamei, Tomoatsu Mune, Kohei Kaku

**Affiliations:** Division of Diabetes, Metabolism and Endocrinology, Kawasaki Medical School, 577 Matsushima, Kurashiki, 701-0192 Japan

**Keywords:** Hypothalamic hypopituitarism, Hypoglycemia, Hydrocortisone

## Abstract

**Introduction:**

Hypothalamic hypopituitarism is relatively rare cause of secondary adrenal deficiency which is often accompanied by severe hypoglycemia. Hydrocortisone replacement therapy is essential for this condition, but gastrointestinal symptom such as nausea and vomiting is not well-recognized adverse effect of hydrocortisone.

**Case description:**

A 64-year-old-woman was referred to our hospital because of frequent severe hypoglycemia. She was diagnosed as type 2 diabetes when she was 58 years old but had not been treated since she was 60. We ruled out the possibility of exogenous administration of insulin or other anti-diabetic drugs, insulinoma and insulin autoimmune syndrome. After glucose injection, she once became conscious, but severe hypoglycemia was often observed even after that. In addition, counter-regulatory hormone levels were not increased even at the time of hypoglycemia. We conducted several hormone load tests. In corticotropin-releasing hormone (CRH) load test, excess and delayed reaction of ACTH was observed. In thyrotropin-releasing hormone (TRH) load test, TSH and prolactin were normally secreted in response to TRH. In luteinizing hormone-releasing hormone (LHRH) load test, delayed reaction of LH and FSH was observed. Based on such hormone loading tests, we diagnosed this patient as idiopathic hypothalamic hypopituitarism and consequent adrenal deficiency. We immediately intravenously injected hydrocortisone and started oral hydrocortisone therapy. However, just after taking hydrocortisone, vomiting was often observed which disturbed sufficient steroid hormone replacement, leading to recurrent hypoglycemia. Therefore, we stopped hydrocortisone and instead started an alternative treatment with prednisolone. After that, vomiting and hypoglycemia were not observed at all.

**Discussion and Evaluation:**

We diagnosed this subject as hypothalamic hypopituitarism mainly by the following two findings: (1) excess and delayed reaction of ACTH in CRH load test, (2) delayed reaction of LH and FSH in LHRH load test.

**Conclusions:**

We should be aware of the possibility of hypothalamic hypopituitarism as a cause of recurrent severe hypoglycemia. Also, we should be aware that hydrocortisone could induce gastrointestinal symptom and that in such a case we should stop hydrocortisone and start prednisolone to sufficiently replace steroid hormone and avoid recurrent hypoglycemia.

## Background

It is well known that hypoglycemia is induced by various reasons such as the overuse of anti-diabetic drugs and/or insulin, various diseases including insulinoma, insulin autoimmune syndrome and adrenal insufficiency. Hypoglycemia influences quality of life and increases the risk of cardiovascular events (Frier et al. 2011; Barendse et al. 2012). When subjects suffer from hypoglycemia, they usually notice it with warning symptoms such as cold sweating and/or palpitation, but recurrent hypoglycemia can lead to the impairment of counter-regulatory hormone response and awareness of hypoglycemia which sometimes leads to life-threatening hypoglycemic coma (Widom and Simonson 1992; Kinsley et al. 1995; Lingenfelser et al. 1995; Kaneto et al. 1998). Hypothalamic hypopituitarism is relative rare cause of adrenal deficiency. Consequent adrenal deficiency is often accompanied by severe hypoglycemia. Hydrocortisone replacement therapy is essential for this condition, but gastrointestinal symptom such as nausea and vomiting is not well-recognized adverse effect of hydrocortisone. Here we report the case of hypothalamic hypopituitarism accompanied by recurrent severe hypoglycemia.

## Case report

Here we report the case of a 64-year-old-woman with hypothalamic hypopituitarism accompanied by recurrent severe hypoglycemia. She was diagnosed as type 2 diabetes in the preoperative examination prior to cataract surgery when she was 58 years old. After then she was treated with insulin for two years, but she did not use any anti-diabetic drugs since she was 60. Since she had experienced severe hypoglycemia many times, she was referred to our hospital to examine the pathogenesis of severe hypoglycemia. On admission, her body weight was 58.9 kg and height was 145cm (Body Mass Index was 28.0 kg/m^2^). There was no abnormality in the chest and abdomen and no pretibial pitting edema. She had no history of traumatic head injury. Furthermore, she had no history of risky jobs and the martial arts, especially boxing and full contact Karate. Blood pressure was 155/71 mmHg and pulse rate was 88 bpm. Neurologic examination showed no abnormality. She did not have visual disturbance and headache. Deep tendon reflex was normal in bilateral lower extremities. Tables 1 and 2 show the laboratory findings on admission. HbA1c was 5.4%, and fasting plasma glucose was 115 mg/dl. Serum insulin level was 1.1 μU/ml, and anti-insulin antibody was 3.8%. Electrolytes, whole blood count, renal and liver function were normal (Table 1). In addition, there was no findings that made us suspect any malignancy, auto-immune and/or inflammatory disease (Table 2). Based on these findings, we ruled out the possibility of exogenous administration of insulin or other anti-diabetic drugs, insulinoma and insulin autoimmune syndrome. After glucose injection, she once became conscious, but severe hypoglycemia was often observed even after that. In addition, counter-regulatory hormone levels were not increased even at the time of hypoglycemia (Table 3).

**Table 1:** **Laboratory findings on admission**

RBC	441 × 10^4^ /μl	TP	7.4 g/dl	CRP	0.13 mg/dl
Hb	13.0 g/dl	Alb	4.3 g/dl	Total chol	169 mg/dl
WBC	8030 /μl	Glb	3.1 g/dl	TG	102 mg/dl
Neu	65.0 %	T-bil	0.5 mg/dl	HDL chol	36 mg/dl
Eos	5.4 %	AST	28 IU/l	LDL chol	109 mg/dl
Baso	0.6 %	ALT	51 IU/l	FPG	115 mg/dl
Mono	6.6 %	γ-GTP	12 IU/l	IRI	1.1 μU/ml
Lymph	22.4 %	LDH	496 IU/l	C-peptide	3.8 ng/ml
plt	34.6 × 10^4^ /μl	ALP	399 IU/l	HbA1c	5.4%
Na	140 mEq/l	ChE	35 IU/l	GA	12.1%
K	3.9 mEq/l	Cre	0.47 mg/dl	anti-insulin Ab	3.8%
Cl	103 mEq/l	BUN	8 mg/dl	anti-GAD Ab	<1.3 U/ml
Ca	9.2 mg/dl	UA	3.8 mg/dl	urine protein	<10 mg/dl
P	3.2 mg/dl	Amy	17 U/l	urine albumin	20.3 mg/gCr

**Table 2:** **Laboratory findings on admission**

ACTH	29.5 pg/ml	TSH	4.19 μΙU/ml	CEA	3.4 ng/ml
cortisol	8.9 μg/dl	FT3	2.92 pg/ml	CA19-9	18 U/ml
plasma renin activity	1.7 ng/ml/h	FT4	0.76 ng/dl	CA125	19.9 U/ml
aldosterone	55.7 pg/ml	anti-Tg Ab	25.2 U/ml	AFP	3.1 ng/ml
DHEA-S	123 μg/dl	anti-TPO Ab	9.6 U/l	PIVKA-II	23 mAU/ml
LH	24.0 mU/ml	TRAb	1.6 U/l	DUPAN	≤25U/ml
FSH	66.9 mU/ml	TSAb	140%	SPAN-1	7.5 U/ml
PRL	18.1 ng/ml	anti-nuclear antibody	(-)	CYFRA	≤1.0 ng/ml
GH	0.09 ng/ml	pituitary cell antibody	(-)	SCC	0.9 ng/ml
somatomedin C	114 ng/ml	IgG4	<3.0 mg/dl	ProGRP	46.5 pg/ml
adrenaline	23 pg/ml	1,25-(OH)2 vitaminD	63.4 pg/ml	QFT	(-)
noradrenaline	240 pg/ml	urine Na	42 mEq/l	Tb Ab	<0.05 U/ml
dopamine	15 pg/ml	urine K	13 mEq/l	ACE	12.3 U/l
				CMV pp65 Ab	(-)

**Table 3:** **Various counter-regulatory hormone levels at the time of hypoglycemia**

PG (mg/dl)	42	36
IRI (μU/ml)	<1.0	<1.0
C-peptide (ng/ml)	0.1	0.1
glucagon (pg/ml)	75	117
ACTH (pg/ml)	16.4	10.9
cortisol (μg/dl)	4.6	5.6
GH (ng/ml)	0.59	1.09
adrenaline (pg/ml)	40	40
noradrenaline (pg/ml)	287	489
dopamine (pg/ml)	17	41

**Figure 1:**
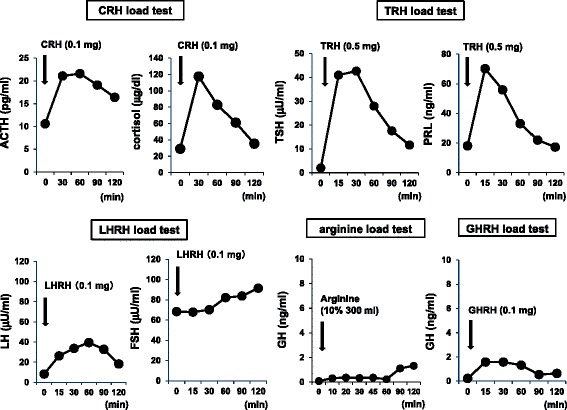
**Corticotropin-releasing hormone (CRH) load test: after intravenous injection of CRH (0.1 mg), ACTH and cortisol levels were examined.** Thyrotropin-releasing hormone (TRH) load test: after intravenous injection of TRH (0.5 mg), TSH and PRL levels were examined. Luteinizing hormone-releasing hormone (LHRH) load test: after intravenous injection of LHRH (0.1 mg), LH and FSH levels were examined. Arginine load test: after intravenous injection of arginine (10%, 300ml), GH level was examined. Growth hormone releasing hormone (GHRH) load test: after intravenous injection of GHRH (0.1 mg), GH level was examined.

To explore the pathogenesis of severe hypoglycemia, we performed several examination. First, magnetic resonance imaging (MRI) of the brain showed no obvious abnormalities; pituitary abnormality such as adenoma and pituitary stalk interruption was not observed. Second, we conducted several hormone load tests (Figure 1). In corticotropin-releasing hormone (CRH) load test, excess and delayed reaction of ACTH (adrenocorticotropic hormone) was observed, indicating the dysfunction of the hypothalamus. In thyrotropin-releasing hormone (TRH) load test, TSH and prolactin were normally secreted in response to TRH. In luteinizing hormone-releasing hormone (LHRH) load test, delayed reaction of LH and FSH (follicle stimulating hormone) was observed, which was also compatible with the possible dysfunction of the hypothalamus. Furthermore, in arginine load test, GH reaction was very poor, which strengthened the idea that the hypothalamus in this subject does not function well. Based on these findings, we diagnosed this subject as hypothalamic hypopituitarism although its cause remained unknown. In growth hormone-releasing hormone (GHRH) load test, GH reaction was poor. Although the results in this GHRH load test suggest the possibility of adult GH deficiency, serum IGF-1 (insulin-like growth factor-1) level was within normal range. Therefore, we followed up without any medication for this poor GH response.

To treat this hypothalamic hypopituitarism and consequent adrenal deficiency, we intravenously injected hydrocortisone (100 mg) and started oral hydrocortisone therapy (10 mg/day). However, just after taking hydrocortisone, vomiting was often observed. Since gastrointestinal symptom was not common adverse effect of hydrocortisone, we suspected the acute gastric ulcer and/or gastritis. Then we performed the gastroesophageal endoscopy, but no abnormalities was found. Therefore, we continued to try to replace steroid hormone with hydrocortisone. However, it seemed that such repeated vomiting disturbed sufficient steroid hormone replacement, leading to recurrent hypoglycemia. Several days later, we gave up the therapy with hydrocortisone and instead started an alternative treatment with prednisolone (15 mg/day) to replace steroid hormone. After that nausea and vomiting disappeared and hypoglycemia was not observed at all.

## Discussion and evaluation

In this report, we showed the subject with hypothalamic hypopituitarism which was diagnosed based on the following findings: excess and delayed reaction of ACTH in CRH load test, delayed reaction of LH and FSH in LHRH load test, poor GH reaction in arginine load test. Although we understood that it would be important to perform insulin tolerance test in order to reconfirm the dysfunction of the hypothalamus in this subject, we failed to obtain the agreement from this subject about the insulin load test due to the risk of the occurrence of hypoglycemia. It was reasonable that this subject was very afraid of the occurrence of hypoglycemia because she experienced severe hypoglycemia repeatedly. Therefore, we decided not to perform insulin tolerance test in this subject. The GH releasing hormone + arginine (GHRH + ARG) test is the best method to accurately evaluate GH secretion. However, according to the Japanese guideline for adult GH deficiency (The hypothalamic-pituitary dysfunction study group of the Ministry of Health Labour and Welfare, Japan 2009), we performed arginine load test without GHRH. Therefore GH response should be considered as low because of insufficient stimulation of GH secretion. However, based upon the other various findings, we thought that such recurrent severe hypoglycemia in this subject was presumably due to hypothalamic hypopituitarism.

We ruled out the possibility of malignancy, sarcoidosis, tuberculosis and traumatic injury, all of which could be the reason for hypothalamic hypopituitarism (Sharma and Sharma 1991; Saito et al. 2009; Greco 2012; Glezer and Bronstein 2012), and thus we thought that the hypothalamic hypopituitarism in this subject was idiopathic. In addition, counter-regulatory hormone levels were not increased even at the time of hypoglycemia (Table 3). We think that this was presumably due to recurrent hypoglycemia as previously reported (Widom and Simonson 1992; Kinsley et al. 1995; Lingenfelser et al. 1995; Kaneto et al. 1998).

In general, gastrointestinal symptom such as nausea and vomiting is not common adverse effect of hydrocortisone. In addition, prednisolone (15 mg/day) is stronger than hydrocortisone (10 mg/day). Therefore, we cannot exclude the possibility that the gastrointestinal symptom in this subject was due to adrenal insufficiency during the hydrocortisone treatment and that the administration of prednisolone improved adrenal insufficiency which led to the disappearance of gastrointestinal symptom. However, considering the situations that nausea and vomiting appeared just after taking hydrocortisone, we think it is likely that such gastrointestinal symptom is the adverse effect of hydrocortisone although its mechanism remains unknown.

## Conclusions

We should be aware of the possibility of hypothalamic hypopituitarism as a cause of recurrent severe hypoglycemia. In addition, although gastrointestinal symptom is not common adverse effect of hydrocortisone, we should be aware that hydrocortisone could induce several gastrointestinal symptom such as nausea and/or vomiting. Such symptom disturbs sufficient steroid hormone replacement and leads to recurrent hypoglycemia. Therefore, in such a case we should stop hydrocortisone and instead start an alternative treatment with prednisolone without any hesitation in order to sufficiently replace steroid hormone and to avoid recurrent hypoglycemia.

## Consent

Informed consent was obtained from this patient for being included in the case report.

### Statement of Human and Animal Rights

All procedures followed were in accordance with the ethical standards of the responsible committee on human experimentation (institutional and national) and with the Helsinki Declaration of 1975, as revised in 2008 (Lingenfelser et al. 1995).

## Abbreviations

RBC: Red blood cell
Hb: Hemoglobin
WBC: White blood cell
Neu: Neutrophil
Eos: Eosinophil
Baso: Basophil
Mono: Monocyte
Lymph: Lymphocyte
plt: Platelet
TP: Total protein
Alb: Albumin
Glb: globulin
T-bil: Total bilirubin
AST: Aspartate transaminase
ALT: Alanine transaminase
ɣ-GTP: amma-glutamyl transferase
LDH: Lactate dehydrogenase
ALP: Alkaline Phosphatase
ChE: Cholinesterase
Cre: Creatinine
BUN: Blood urea nitrogen
UA: Uric acid
Amy: Amylase
CRP: C-reactive protein
Total chol: Total cholesterol
TG: Triglyceride
HDL chol: High density lipoprotein cholesterol
LDL chol: Low density lipoprotein cholesterol
FPG: Fasting plasma glucose
IRI: Imunoreactive insulin
HbA1c: Hemoglobin A1c
GA: Glycoalbumin
anti-GAD Ab: Anti-glutamic acid decarboxylase antibody
ACTH: Adrenocorticotropic hormone
DHEA-S: Dehydroepiandrosterone sulfate
LH: Luteinizing hormone
FSH: Follicle stimulating hormone
PRL: Prolactin
GH: Growth hormone
IGF-1: Insulin-like growth factor-1
TSH: Thyroid*-*stimulating hormone
FT3: Free triiodothyronine
FT4: Free thyroxine
anti-Tg Ab: Anti-thyroglobulin antibody
anti-TPO Ab: Anti-thyroid peroxydase antibody
TRAb: TSH-receptor autoantibody
TSAb: Thyroid- stimulating autoantibody
IgG4: Immunoglobulin G4
CEA: Carcinoembryonic antigen
CA19-9: Carbohydrate antigen 19–9
CA125: Cancer antigen 125
AFP: Alpha-fetoprotein
PIVKA-II: Proteins induced by vitamin K absence
DUPAN-2: Duke pancreatic monoclonal antigen type 2
SPan-1: s-pancreas-1 antigen
CYFRA: Cytokeratin fragment
SCC: Squamous cell carcinoma
ProGRP: Pro gastrin-releasing peptide
QFT: QuantiFERON
Tb Ab: *tuberculosis* antibody
ACE: Angiotensin-converting enzyme
CMV pp65 Ab: Anti-Cytomegalovirus pp65 antibody
